# Evolution of cellular morpho-phenotypes in cancer metastasis

**DOI:** 10.1038/srep18437

**Published:** 2015-12-17

**Authors:** Pei-Hsun Wu, Jude M. Phillip, Shyam B. Khatau, Wei-Chiang Chen, Jeffrey Stirman, Sophie Rosseel, Katherine Tschudi, Joshua Van Patten, Michael Wong, Sonal Gupta, Alexander S. Baras, Jeffrey T. Leek, Anirban Maitra, Denis Wirtz

**Affiliations:** 1 Johns Hopkins Physical Sciences – Oncology Center, The Johns Hopkins University, Baltimore, Maryland 21218, USA; 2 Department of Chemical and Biomolecular Engineering, The Johns Hopkins University, Baltimore, Maryland 21218, USA; 3 Department of Pathology, Johns Hopkins University School of Medicine, Baltimore, Maryland 21231, USA; 4 Department of Pathology, UT MD Anderson Cancer Center, Houston, Texas, 77030, USA; 5 Department of Oncology, Johns Hopkins University School of Medicine, Baltimore, Maryland 21231, USA; 6 Department of Pathology and the Sol Goldman Pancreatic Cancer Research Center, Johns Hopkins University School of Medicine, Baltimore, Maryland 21231, USA; 7 Department of Biostatistics, Johns Hopkins Bloomberg School of Public Health, Baltimore, MD, 21205, USA

## Abstract

Intratumoral heterogeneity greatly complicates the study of molecular mechanisms driving cancer progression and our ability to predict patient outcomes. Here we have developed an automated high-throughput cell-imaging platform (htCIP) that allows us to extract high-content information about individual cells, including cell morphology, molecular content and local cell density at single-cell resolution. We further develop a comprehensive visually-aided morpho-phenotyping recognition (VAMPIRE) tool to analyze irregular cellular and nuclear shapes in both 2D and 3D microenvironments. VAMPIRE analysis of ~39,000 cells from 13 previously sequenced patient-derived pancreatic cancer samples indicate that metastasized cells present significantly lower heterogeneity than primary tumor cells. We found the same morphological signature for metastasis for a cohort of 10 breast cancer cell lines. We further decipher the relative contributions to heterogeneity from cell cycle, cell-cell contact, cell stochasticity and heritable morphological variations.

Pancreatic ductal adenocarcinoma (PDAC), is one of the most devastating human malignancies, it is characterized by extensive local invasion, early systemic dissemination, and pronounced resistance to chemotherapy and radiotherapy^1^. Five-year survival rates for patients diagnosed with invasive pancreatic cancer is <3%^2^,^3^,^4^,^5^,^6^,^7^. Multiple studies have been conducted to investigate the molecular mechanisms of tumorigenesis for pancreatic cancer^8^,^9^,^10^,^11^. The recent sequencing of the PDAC genome by Jones *et al.* confirmed that the majority of patients harbor mutations in one of four genetic “mountains” - *KRAS, TP53, CDKN2A/p16* and *SMAD4/DPC*^8^. Confounding this genetic landscape, however, was the fact that beyond these four “mountains”, one finds a plethora of low-frequency somatic mutations (“hills”), which greatly adds to the complexity of the PDAC genome. This confounding genetic landscape is in part a result of ongoing cancer cell evolution driven by genomic instability^9^,^12^ and cellular heterogeneity^12^,^13^,^14^. Even though accumulating evidence indicates that metastatic tumors are established by sub-clones of primary tumors^8^,^9^, non-consensus genomic profiles displayed by metastatic tumors greatly limit the ability of genetic profiling to assess tumors and predict clinical outcomes^8^,^15^.

To metastasize, a cell must overcome multiple obstacles in the metastatic cascade^16^ - invasion and migration through the dense, tortuous stromal matrix^17^; intravasation^18^, survival from shear forces of blood flow^19^, successful re-attachment to blood vessel walls^16^,^19^– are directly associated with the physical properties of cells^20^. Thus, cell physical properties and cell phenotypic profiles are likely deterministic descriptors of metastasis. In fact, highly metastatic cells often show a mechanically softer cytoplasm compared to non-metastatic cells in many types of cancers^21^. Furthermore, various cell line model systems demonstrate common changes in physical properties, such as traction forces, migratory behavior, and mechanical stiffness^22^,^23^,^24^,^25^. However, these studies do not account for patient-to-patient variations, thereby the role of heterogeneity that is considered a hallmark of cancer has not been fully addressed^8^,^19^,^26^,^27^.

To determine the role of cell physical properties and its association between tumor evolution and metastasis, we investigated the morphology of multiple different PDAC cell lines with fully sequenced exomes^8^. The cell lines originated from either the primary site in the pancreas or from metastatic sites, mainly in the liver. We developed a high-throughput machine-vision system to rapidly record and analyze the morphology of thousands of cells, which we call visually-aided morpho-phenotyping recognition (VAMPIRE). VAMPIRE analysis allows us to classify irregular cellular and nuclear shapes and provide an effective visual aid to display and compare these shapes. Several thousand individual cells are characterized in <20 min for each cell line. We show that cell morphology is a complex product of cell cycle, local cell density, cell stochasticity and heritable cell variations. We found that the primary tumor cell lines present significantly higher heterogeneity and heritable cell variations in cell and nuclear shape compared to cells derived from metastatic sites. We found the same relation among 10 tested breast cancer cells lines. Together, our results provide evidence at the cell phenotypic level that metastasis arises through clonal selection, and indicate that cellular physical properties play an important role in cancer progression.

## Results

### The htCIP assay and VAMPIRE analysis

**Figure 1:**
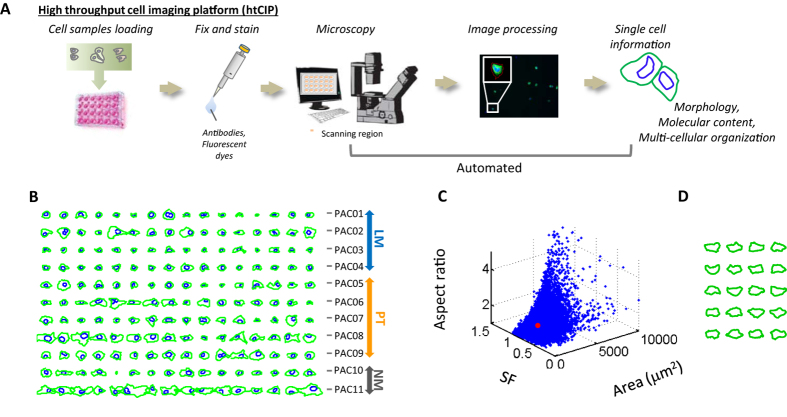
High-throughput cell imaging platform (htCIP) and morphology of PDAC cells. (**A**) Cell samples were seeded on 24-well glass plates and then fixed and stained. Images were acquired on an automated-stage epifluorescence microscope using a standardized scanning grid. Each fluorescence channel was subsequently processed using custom software and data was then extracted for analysis. (**B**) Sixteen randomly chosen, horizontally aligned cell and nuclear traces from each patient-derived pancreatic cancer cell line shown here for qualitative visual comparison. (**C**) Three-dimensional scatter plot showing the wide spectrum of conventional morphological descriptors, cell size, shape factor (SF) and cell aspect ratio, for the pancreatic cancer cell lines used in this study (n = 11 samples and 39,000 individual cells). (**D**) This panel shows that even for highly similar values of cell size, SF and cell aspect ratio represented by range of red spot in panel (**C**), the corresponding cells can still display a wide range of shapes not captured by these conventional morphological descriptors.

We developed a high-throughput cell imaging platform (htCIP) that allowed us to extract high-content information for individual cells, including cellular and nuclear morphology, molecular content, and local multi-cellular organization (see schematic in Fig. 1A and more details in supplementary information). A low-magnification, low numerical aperture objective was used in this assay, which allowed for rapid imaging of a large number of individual cells. The cellular and nuclear shape of fluorescent-labeled individual pancreatic cancer cells on glass bottom plates was segmented and analyzed using a custom image processing software. Detailed experimental and computational procedures are given in SI Experimental Procedures. We validated the cell shapes extracted by this assay by comparing the results obtained from automated segmentation and manual tracing (Figs S1A and S1B). We verified that the use of this low-magnification objective had sufficient optical resolution for measuring cellular and nuclear features by comparing results obtained using low- and high-magnification lenses (Fig. S1C). We used this assay to identify a potential morphological signature of metastasis in pancreatic ductal adenocarcinoma (PDAC) using nine previously sequenced^8^, patient-derived, primary tumor (PT; five lines) and liver metastatic (LM; four lines) cell lines. In addition, two distinct non-neoplastic pancreatic epithelial cell lines (NM) were included for cross comparison (Table S1).

We analyzed the shapes of thousands of individual cells and their nuclei. For direct visual assessment of cell and nuclear shapes, rotationally-invariant shapes of cells and associated nuclei were obtained by aligning the major axis of the cell/nucleus outlines along the horizontal axis. (see more details in Materials and Methods). Randomly selected subsets of individual cell traces did not reveal overt morphological differences between PT and LM cells, presumably due to the irregularity of cell shapes (Fig. 1B). Morphological features, such as spreading area, shape factor, and aspect ratio, have been widely used to describe cell shape, yet, these features could not reflect the extent of cell shape variations, since even a small subset of cells displaying an extremely narrow range of values of these conventional shape descriptors appeared radically different from each other (Fig. 1C,D).

**Figure 2:**
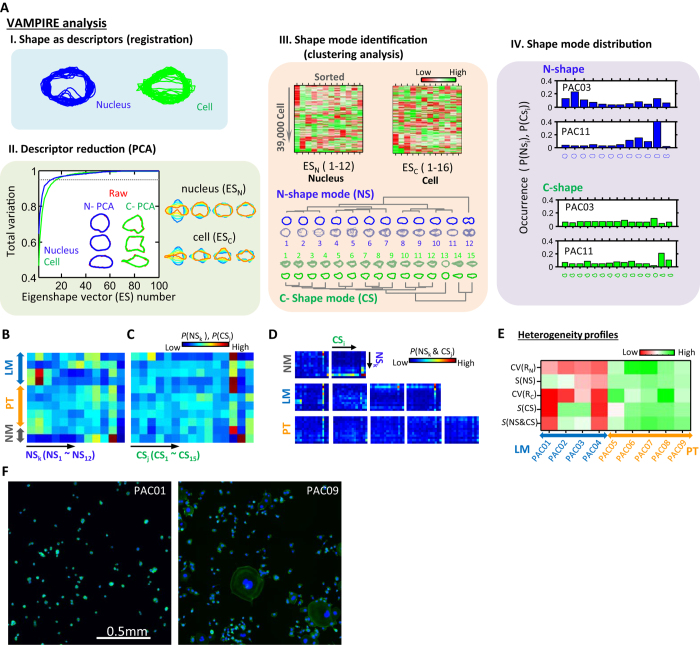
Visually-aided morpho-phenotyping recognition (VAMPIRE) analysis. (**A**) Demonstration of VAMPIRE analytical processes. (**B–D**) Heat maps show the probability of cells in each nucleus and cell shape modes (*P*(NS_k_) or *P*(CS_k_)) for each different sample (**B**,**C**). Heat maps show the population distributions of different paired nuclear and cellular shapes modes (*P*(NS_k_&CS_j_)) for different samples (**D**). Color coding (blue to red) corresponds to low and high occurrence. (**E**) A panel of five heterogeneity properties, including CV of nucleus (CV(R_N_)) and cell size (CV(R_C_)), entropy of nucleus (*S*(NS)) and cell shape (*S*(CS)), and entropy of paired nucleus-cell shape (*S*(NS&CS)) was used to represent overall heterogeneity profiles of cell morphology of 9 PT and LM samples. The magnitude of heterogeneity of these cell populations is shown in a heat map where color from red to green indicates increasing degree of heterogeneity. (**F**) Images of PAC01 cells and PAC09 cells. Nuclei and F-actin are labeled in blue and green.

To address this problem, we developed the VAMPIRE assay, which analyzes irregular cellular and nuclear shapes and provides a visual aid for the direct comparison of cell morphologies (Fig. 2A). The VAMPIRE assay identifies representative shape modes among cell shapes presented by all cells and determines the occurrence of these shape modes for large cell populations. VAMPIRE analysis comprises four essential steps: I) the determination of the coordinates of equally-spaced points along the nuclear and cellular shapes; II) the reduction of the number of morphological descriptors using principal component analysis (PCA); III) the identification of shape modes, and IV) the analysis of shape mode distributions. To represent the infinite number of possible cell shapes, we used 50 points (i.e. 100 coordinates) equally spaced along the periphery of any given cell, defined here as “features”. As previously demonstrated^28^,^29^,^30^,^31^, any cell shape can be represented by a limited number of eigenshapes determined by PCA applied to all cell features (100 times the total number of cells for all conditions, i.e. 100 × 39,000). The scaling factor (*R*) of a given cellular or nuclear shape was computed to represent cell and nucleus size (see definition in supplementary information) and used to unify the scale of all analyzed shapes, eliminating the confounding effects of cell and nuclear size. An alignment procedure was then used to eliminate the effects of rotational variations in the PCA (Fig. 2A, upper left). The projection scores of nucleus shapes and cell shapes on eigenshape vectors that comprise 95% of variations were used to represent cell shapes.

We found that 95% of shape variations for all nuclei and cells were captured by just 12 and 16 eigenshape vectors, respectively. This result further confirmed that the shape factor or the aspect ratio of a cell (a single parameter) was insufficient to accommodate the observed large variations in nuclear and cellular morphology. As a proof of concept, we were able to accurately reconstruct the experimentally determined morphologies of randomly selected cells using the 12 and 16 eigenshape vectors, respectively (Fig. 2A, bottom left).

Eigenshape vectors are mathematically defined and data-driven. Even though their association with morphology can be graphically represented, their underlying biological meaning is difficult to illustrate. Therefore, we further implemented a K-means clustering analysis to empirically identify representative morphological subtypes among these cells. From our dataset of over 39,000 cells encompassing all the studied PDAC samples, cells and nuclei were categorized into 12 different modes for nuclear shapes and 15 different modes for cellular shapes (Fig. 2A, middle panel). The representative morphology of each shape mode was reconstructed using cluster-centroid values at different eigenshape vectors. The number for shape modes was estimated using the separation index and the Xie and Beni index (Fig. S2)^32^,^33^. Corresponding shape modes for each individual cell were assigned and the distribution profiles of cellular and nuclear shape modes from PDAC cell samples were examined, thus revealing unique signatures for different PDAC cell samples (Fig. 2A, right panel (IV)). The robustness of this analysis was confirmed by reproducing results from replicate biological samples (Fig. S3).

### Cell morphology signature for metastatic potential

Analysis of shape mode distributions demonstrated that a morphological phenotype could sometimes be shared between two different PDAC cell samples (Fig. 2B,C and S4). However this similarity was not shared across all PT or all LM samples. Instead, we found that PT cells displayed a more uniform distribution of nuclear shape modes than LM cells. The same trend held in paired nucleus-cell shape mode distributions (Fig. 2D). This suggested that metastatic potential was associated with cell morphological heterogeneity.

Thus, we evaluated the heterogeneity profiles of PDAC samples using the coefficient of variance (CV) of nucleus and cell size, the “Shannon entropy” of nucleus and cell shape mode distributions, and the entropy of nucleus-cell paired shape mode distributions (Fig. 2E). This analysis revealed a clear pattern of significantly elevated morphological heterogeneity among PT samples in contrast to LM samples (Fig. 2F and Fig. S5A-S5E). Of note, this different degree of heterogeneity in shapes was not detectable when using “conventional” morphological descriptors (Fig. S5F-S5I). This morphological heterogeneity was similarly not reflected in the variations in the number of somatic mutations in PT and LM cells (Fig. S6). We estimate that, on average, the number of altered genes in LM cell lines was only slightly higher but not significantly (*P* = 0.40) compared to PT cell lines. Furthermore, we found that breast cancer cells derived from metastatic sites also exhibited lower heterogeneity relative to cells derived from primary tumors (Fig. S7 and Table S2). Together, our results suggest that cell dissemination from a primary tumor to distant locations is associated with the loss in cell morphological heterogeneity.

### Cell heterogeneity in 3D environments

**Figure 3:**
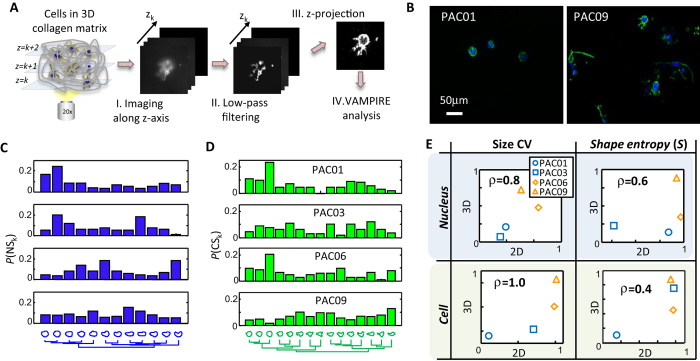
Cellular heterogeneity in 2-D and 3-D environments. (**A**) Strategy to analyze cell morphology in 3D collagen matrices. Z-stack images are obtained through sequential imaging at different z positions. A low-pass filter is then applied to individual images followed by maximum z-projection. Cell morphology in projected image is then obtained and subjected to VAMPIRE analysis. (**B**) Images of PAC01 cells and PAC09 cells in 3-D collagen matrices after z-projection. Nuclei and F-actin are labeled in blue and green. (**C,D**) Nucleus shapes modes and cell shape modes are identified by VAMPIRE analysis for cells in 3-D matrices. Histograms show nuclear and cellular shape mode distributions for PAC01, PAC03, PAC06 and PAC09 cells. More than 100 cells were analyzed for each sample. (**E**) Cell morphological heterogeneity properties in both 2D and 3D environments. Strong positive correlation is observed for CV of nucleus size (Spearman’s correlation coefficient ρ=0.8), CV of cell size (ρ=1.0), shape entropy of nucleus (ρ=0.6) and shape entropy of cell (ρ=0.4).

We next studied whether the difference in morphological heterogeneity between LM and PT cells held when these cells were fully embedded in 3D collagen matrices, a more physiological relevant condition^34^,^35^,^36^,^37^,^38^. Therefore, we extended our methodology to analyze cell morphology in 3D matrices (Fig. 3A). To reduce complexity, we analyzed projected 2D images from z-stack image sets. Cell shapes in the projected images were then extracted and subjected to VAMPIRE analysis. We applied this analysis to four PDAC samples, which exhibited different morpho-phenotypes (Fig. 3B). As expected, cells in 3D matrices displayed more irregular shapes than the same cells placed on 2D substrates (Fig. 3C,D). However, the distribution of shape modes in PT cells remained more heterogeneously distributed than LM cells. Morphological heterogeneity in nuclear shape, nuclear size, cell shape, and cell size for cells on 2D substrates strongly correlated with those in 3D collagen matrices (Fig. 3E). Hence, data suggests that morphological heterogeneity is a cell-intrinsic signature that is independent of the “dimensionality” of the cellular environment.

### Cell morphology dependent on cell cycle and local cell density

**Figure 4:**
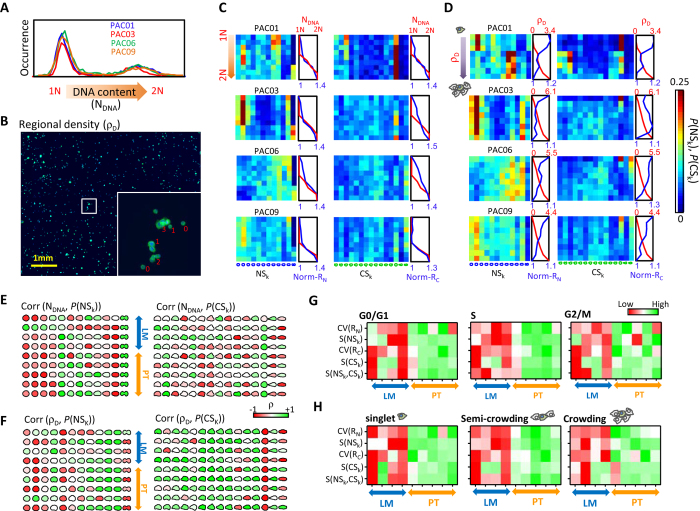
Effects of cell cycle and local cell density on cell heterogeneity. (**A,B**) htCIP provides versatile single-cell measurements including cell DNA content (**A**) and local cell density (ρ_D_) (**B**), for cells on 2D substrates. (**C,D**) Relation between cell morphology and progression of cell cycle (**C**) and increase in local cell density (**D**) for PAC01, PAC03, PAC06 and PAC09. Cells were sorted based on their DNA content and divided into 9 groups with equal sample size. Probability of cells found in different nucleus and cell shape modes (P(NS_k_), P(CS_k_)) at these groups are shown in heat maps. The averaged DNA contents, normalized nucleus size and normalized cell size for these groups are shown in the plot next to the heat maps. Nucleus and cell size are normalized by dividing the lowest value among all groups. The same procedures are used to show effect of local cell density on cell morphology. (**E,F**) Nucleus and cell shape modes, filled with colors to show correlation between DNA content and occurrence of individual shape modes (**E**). Color coding from red to green corresponds to Pearson’s correlation coefficient from −1 to 1. The same procedure is applied to show correlation between local cell density and occurrence of shape modes (**F**,**G**) Overall heterogeneity profiles of cell morphology of PAC samples in the G_0_/G_1_, S and G_2_/M phases. (**H**) Overall heterogeneity profiles of cell morphology of PAC samples under three local density conditions, including singlet (ρ_D_ = 0) semi-crowded (0 < ρ_D_ < 4), and crowded (ρ_D_ ≥ 4). The heterogeneity of these samples is shown in a heat map where colors from red to green indicate an increasing degree of heterogeneity.

Progression through the cell cycle increases cell size and modulates cell shape^39^,^40^,^41^. In addition, PDAC cells form cell-cell contacts and ductal-like structures^42^. Hence, cell cycle and cell-cell contacts could influence morphological heterogeneity. The htCIP assay also provides accurate intensity measurements and multi-cellular status at the single-cell level and allows for the direct investigation of the association of cell morphology with cell cycle and local cell density^43^. First, we ensured that DNA content distribution measured by htCIP and standard flow cytometry were similar (Fig. 4A). Next, we computed the precise locations of cells, which enabled us to extract cell-cell contact information, without losing counts of cell-cell contacts for cells that were at the boundaries of individual images (Fig. 4B).

By combining information on cell cycle and cell-cell contacts with VAMPIRE analysis, we analyzed how cell morphology depended on cell cycle and local cell density (Fig. 4C,D). In general, cell and nuclear sizes increased simultaneously with an increase in DNA content and a decrease in local cell density. The distribution of shape modes was also dependent on cell cycle phase and local density. The fractions of cells in various shape modes (*P*(NS_k_), *P*(CS_k_)) displayed some degree of positive (green color) or negative (red color) correlation with DNA content and local density (Fig. 4E,F). Furthermore, we found that the difference in heterogeneity between LM and PT cells did not result from effects of cell cycle or local cell density since this difference remained when restricting our analysis to cells in any specific cell cycle phase (either G_0_/G_1_, S or G_2_/M) or specific density conditions (Fig. 4G,H). However, the heterogeneity differences between LM and PT cells were generally stronger for specific clustered cells compared to overall cell populations (Table S3).

**Figure 5:**
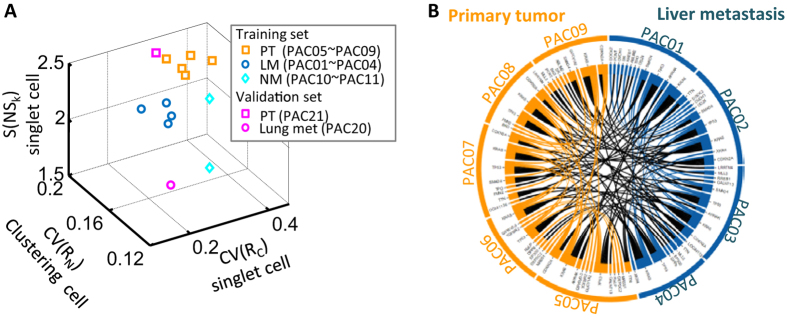
Predictive signature for metastatic pancreatic cancer cells. (**A**) A 3-D scatter plot shows delineation between different types of samples using CV of nuclear size distribution among cell clusters, CV of cell size distribution among singlet cells and entropy of nucleus shape among single cells for each sample. Three distinct subgroups, respectively composed of PT, LM and NM samples, are readily observed. Two additional patient-derived pancreatic cancer cell lines, a primary tumor cell line (PAC21) and a lung metastases cell line (PAC20), were introduced as validation samples and these two parameters were measured. The location of these two parameters from the new primary tumor derived cell line overlayed well with the PT cluster previously obtained with the training set. The cell line derived from lung metastatic region co-clusters with LM cluster previously obtained with the training set. (**B**) Relationship of repeating somatic mutations between PT and LM is represented using a Circos plot that is generated using custom software in *R*. No distinct somatic mutation signature is identified for cells derived from the metastatic site (Interactive Circos plot can be viewed in supplementary materials).

Furthermore, it has been previously shown that metastatic melanoma cells exhibit dynamic phenotypes in response to microenvironmental perturbations^44^. Together, these results suggest that specific cellular and micro-environmental conditions may enhance differences in biophysical properties associated with metastatic potential. The most distinct differences between LM and PT were observed in the CV of cell sizes among singlet cells, the CV of nucleus sizes among clustered cells, and the entropy in nuclear shapes among singlet cells. A three-dimensional plot corresponding to these features showed a clear separation among LM, PT, and NM (Fig. 5A), suggesting that the loss of morphological heterogeneity was a robust metastatic phenotypic signature.

### Validation for the metastatic morphological signature

To further validate this metastatic morpho-phenotypic signature, we analyzed two additional pancreatic cancer cell lines, cells derived from a pancreatic primary tumor (PAC21) and cells derived from a lung metastasis (PAC20). The new PT cell line was located in the proximity of other PT cell lines from the original training dataset, while the cell line derived from the lung metastasis overlapped with training LM cell lines (pink square and circle in Fig. 5A).

We performed a sensitivity analysis of the discrimination between PT and LM cells. We repeated random sub-sampling cross-validation and investigated the accuracy of using these parameters to predict the metastatic status. Different sample sizes were tested and the accuracy in predicting metastatic status reached >95% for a sample size of ~300 cells (Fig. S8). Together these results suggest that the lower variation in morphology is predictive of cells derived from metastatic sites. Importantly, among our tested patient-derived cells, no distinctive mutational signature was identified between metastatic and primary cell lines^8^ (Fig. 5B, and see interactive figure at http://biostat.jhsph.edu/~jleek/code/figure2.html). Although most of the PT and LM samples that we tested harbor mutations in *KRAS, TP53, SMAD4* and *CDKN2A*, we did not find an individual mutation or set of mutations that occurred exclusively in either all PT or all LM samples, respectively. This finding indicates that the distinct morphological characteristics of PT cells and LM cells were not directly associated with the occurrence of specific somatic mutations.

### Hierarchy of cell heterogeneity

**Figure 6:**
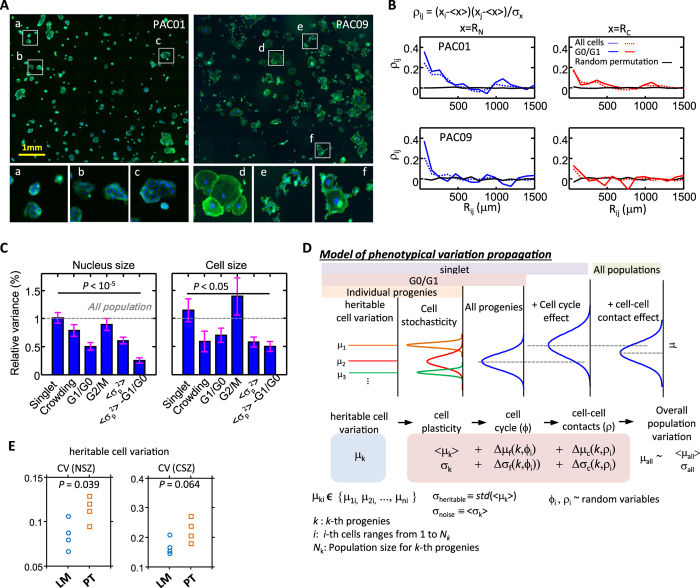
Hierarchy of cell heterogeneity. (**A**) Images of PAC01 and PAC09 cells after four-day growth from a sparse initial seeding density and show cells forms several spatially and morphologically distinct progenies. Detailed view of cell images in highlighted areas are shown in the bottom. (**B**) Paired correlation analysis of nucleus size and cell size for PAC01 and PAC09 cells. Elevated correlation for cells in proximity was found in both all population and cells in G_0_/G_1_ phase. After randomly permutation label cells, this correlation disappears. (**C**) Bar graphs show the average variances of nucleus size and cell size among different pancreatic cancer cells, including PAC01, PAC02, PAC03, PAC04, PAC06, PAC07, PAC08 and PAC09. Variance of cells depends on the underlying cellular conditions including singlet, crowding, in the G_0_/G_1_ phase, in the G_2_/M phase. Averaged nucleus size and cell size variation within a clone (<σ_p_^2^>) for all cells and for cells in the G_0_/G_1_ phase is also shown. The variances are scaled by the variance among all populations. Great decrease in variations in both nucleus size and cell size were found for clonal cells in the G_0_/G_1_ phase. The *P* value is calculated using one-way ANOVA. (**D**) A plot illustrates that observed cell heterogeneity are combination of different effects including cell cycle, cell-cell contact, cell stochasticity and heritable cell variation. A proposed mathematical model to describe cellular heterogeneity is shown in the bottom. (**E**) CV of averaged nucleus size and averaged cell size among different progenies is used to measure heritable variation for both LM (PAC01~PAC04) and PT (PAC06 ~ PAC09). LM display in average lower heritable variation in both nucleus size (*P* < 0.05) and cell size (*P* > 0.05). The *P* value is calculated based on two-sample *t*-test.

Intrinsic morphological variations could be due to stochasticity that is either not passed to progenies or may persist over several generations (heritable cell variations). To study the origin of intrinsic variations displayed by PDAC cells, cells were sparsely placed on substrates and their morphologies were measured after four-day growth. PDAC cells formed several spatially distinct colonies. We found that PDAC cells within different colonies exhibited distinct morphological phenotypes (Fig. 6A).

Pair-wise correlation analysis on cellular and nuclear size showed an elevated correlation for PAC01 and PAC09 cells in close proximity of each other. Since the same trend was found when we sampled cells within the G_0_/G_1_ phase, we conclude that this morphological consistency was not due to cell-cycle synchronization (Fig. 6B). To further understand the origin of morphological heterogeneity, we compared the variance in nucleus size and cell size for cells in different cellular state (i.e. cell cycle) and extracellular conditions (i.e. local cell density) (Fig. 6C). The results showed that singlet cells showed more variance compared to overall cell populations (which contain cell clusters), but cell crowding within clusters greatly reduced this variance. Cells randomly distributed in different cell cycle phases also led to increase in variance. Variance of nucleus and cell size for cells in the G_1_/G_0_ phase was ~50% and ~30% lower than overall population, respectively. However, variance in cell size for cells in the G_2_/M phase was ~40% higher than for the overall population. Importantly, the average variance of nucleus size and cell size for individual progenies for cells in the G_0_/G_1_ phase was ~80% and ~50% lower than the overall population. This decrease in size variance within clonal populations disappeared after random (computer-based) permutation of cells in each experiment (Fig. S9A). Further, we found that nucleus shape, but not cell shape, of PDAC cells within colonies also had significantly lower variance than the overall population (Fig. S9B). This result demonstrates that cellular heterogeneity of PDAC cells result from a combination of cell cycle, cell-cell contacts, heritable cell variations, and cell stochasticity.

Based on these results, we propose a model describing cell heterogeneity based on our results (Fig. 6D). We measured the level of intrinsic cellular variation for LM and PT cells: LM cells display in average lower heritable variation in nucleus size (*P* < 0.05), cell size (*P* > 0.05), nucleus shape and cell shape (Fig. 6E and Fig. S9C). Together, our results suggest that a decrease in intrinsic cell-to-cell variations is strongly associated with metastatic potential.

## Discussion

Automated microscopy and image analysis based on multivariate morphological features is a powerful tool to profile single cells for drug discovery and toxicity predictions^45^,^46^ and to characterize heterogeneous cellular responses^47^. Recent studies utilizing a similar strategy of principal components analysis and unsupervised classification to identify discrete cell shapes for RNAi screen found that gene expression alterations can mediate morpho-phenotypes of cells^44^,^48^,^49^. Here, we analyzed the morphology of cells derived from patients harboring primary tumors and metastases. We demonstrated that direct use of cell traces (i.e. boundary coordinates) after registration can be an effective way to describe complex cell shapes as opposed to the use of conventional morphological descriptors such as cell shape factor and aspect ratio^44^. One primary advantage of the morpho-phenotype analysis proposed in this study is the capability to visualize nuclear and cellular morphologies.

Intraturmoral and intertumoral heterogeneity present not only clinical difficulties, but also obstacles to cancer diagnosis, prognosis and treatment^14^. The study of tumor heterogeneity could have broad impact in cancer management. Our current understanding of tumor heterogeneity in cancer progression stems primarily from studies at the genomic and transcriptomic levels^9^,^13^,^48^,^50^, but little is known at the cellular phenotypic level, and in particular morphology. In this study, we have established that morphological heterogeneity is significantly higher in primary tumors than in metastasized tumor cells, for both pancreatic and breast cancer. This result suggests that metastatic clones derived from subpopulation of a primary tumor that meet the challenges of metastatic barriers, facilitate the phenotypic convergence seen in metastatic samples, which has also been implicated from whole-genome sequencing studies^11^. In our study, we provide quantitative evidence of evolution-selection of cancer metastasis at the cell-phenotypic level. Together, these results suggest that the clonal diversity exhibited within primary tumor populations that stems from their underlying genomic diversities contribute to the convergent morphology observed in metastatic samples.

The analysis of BR04 (MDA-MB-231), a triple negative breast cancer cell line that is derived from a metastasis site, exhibited a high degree of morpho-heterogeneity. Interestingly, all triple negative breast cancer cells (BR04, BR07, BR08 and BR010) consistently displayed a high level of morphological heterogeneity (Fig. S7B). In addition, a recent study that assessed the genomic diversity of breast cancer tissue sections has also shown that that triple negative breast cancer cells exhibit high genomic diversity^51^. Recently, studies have shown that genomic heterogeneity in primary tumors is linked to a worse prognosis in breast cancer and esophageal cancer^52^,^53^. The fact that the study of morphological heterogeneity corresponds well with these genomic studies suggest that the measurement of phenotypic morphological heterogeneity, and functional profiles can be a powerful, high-throughput, and cost-effective platform to diagnose primary tumors compared to single-cell genomic analysis. Since our analysis also demonstrates that morpho-phenotypes of cells can be influenced by their microenvironmental conditions (e.g. cell density), the direct phenotypic analysis of intact tissue sample such as the tissue sections of primary tumors may impose added complications. An alternative way to apply our single-cell analysis for clinical tumor samples would be to harvest cells directly from dissected fresh or frozen tumors, and observe the cellular morphology under uniform microenvironmental conditions to minimize the effects that may be introduced as a function of heterogeneity in microenvironmental conditions^13^. Nevertheless, morphological analysis should be highly compatible with circulation tumor cells samples to profile CTCs heterogeneity^54^,^55^.

It is generally believed that the fastest growing cell clone will eventually dominate cell population with time. Our high-throughput and high-content single cell phenotyping analysis reveals the paradigm of cellular heterogeneity and distinct, heritable cell subtypes in individual cancer cell lines. Cells with different cell morphological subtypes would likely have different cell functions and underlying molecular compositions. Identification and isolation of cell subtypes in a cell line model system can greatly benefit cancer studies that rely primarily on cell line model systems, such as molecular mechanism studies—since the effects of molecular alterations can directly translate into cell functions without complexities due to mixed cell subtypes. It is also imperative to understand the underlying conditions that drive the formation of these distinct cell subtypes that exist in the cell line model, and the evolutionary trajectories of different subtypes. Future work will be needed to elucidate the role of stochastic gene expression or genomic instability, and their causative role in divergent cellular behaviors.

## Additional Information

**How to cite this article**: Wu, P.-H. *et al.* Evolution of cellular morpho-phenotypes in cancer metastasis. *Sci. Rep.* **5**, 18437; doi: 10.1038/srep18437 (2015).

## Supplementary Material

Supplementary Information
