# Supplementary material for: Evolution of cellular morpho-phenotypes in cancer metastasis
Source: Sci Rep. 2015 Dec 17;5:18437. doi: 10.1038/srep18437 (PMC4682070; doi:10.1038/srep18437)
Supplement: Supplementary Information [file srep18437-s1.pdf]

Supplementary information

Evolution of cellular morpho-phenotypes in cancer metastasis

Pei-Hsun Wu[1,2*+], Jude M. Phillip[1,2+], Shyam B. Khatau[1,2], Wei-Chiang Chen[1,2], Jeffrey Stirman[1], Sophie Rosseel[1], Katherine Tschudi[1], Joshua Van Patten[1], Michael Wong[1], Sonal Gupta[4], Alexander S. Baras[3], Jeffrey T. Leek[7], Anirban Maitra[5,6] & Denis Wirtz[1,2,5*]

[1] Johns Hopkins Physical Sciences - Oncology Center, The Johns Hopkins University, Baltimore, Maryland 21218, USA

[2] Department of Chemical and Biomolecular Engineering, The Johns Hopkins University, Baltimore, Maryland 21218, USA

[3] Department of Pathology, Johns Hopkins University School of Medicine, Baltimore, Maryland 21231, USA

[4] Department of Pathology, UT MD Anderson Cancer Center, Houston, Texas, 77030, USA

[5] Department of Oncology, Johns Hopkins University School of Medicine, Baltimore, Maryland 21231, USA

[6] Department of Pathology and the Sol Goldman Pancreatic Cancer Research Center, Johns Hopkins University School of Medicine, Baltimore, Maryland 21231, USA

[7] Department of Biostatistics, Johns Hopkins Bloomberg School of Public Health, Baltimore, MD, 21205, USA

[*]Corresponding authors: Denis Wirtz (wirtz@jhu.edu) and Pei-Hsun Wu (pwu27@jhu.edu)

[+] These authors contributed equally

Experimental procedures

Cell cultures

To keep experimental cell samples in low passage number, a maximum of five passages were allowed after thawing cell samples from deep storage for experiments. All cell lines were cultured at 37^0C and 5% CO_2, approximately 10 days prior to imaging. The culture medium for patient derived pancreatic cancer cells is DMEM (Invitrogen, Carlsbad, CA) with 10% FBS (Gemini Bio-Products, Sacramento, CA). HPDE cells were maintained in Keratinocyte-SFM medium (Invitrogen) with 0.1 ng/ml hEGF (Invitrogen) and 25ul/ml bovine pituary extract (Invitrogen). HPNE cells were maintained in the medium which is composed of 25% low glucose DMEM (Invitrogen), 70% M3 base media (Incell, San Antonio, TX), 5% FBS, 25μg/ml Gentamicin (Quality Biological, Gaithersburg, MD), and 10ng/ml hEGF. Antibiotics were supplemented in all culture medium with the concentration of 100 IU/ml penicillin, and 100μg/ml streptomycin (Sigma-Aldrich, St. Louis, MO). Cell lines were passed every 3 to 4 days, based on their growing conditions. Breast carcinoma cell lines were purchased from ATCC (American type cell culture, Manassas, VA) with authentication done by the provider. Cells (BR01-BR03, BR06-BR11) were cultured on tissue culture dishes in RPMI-1640 medium (Gibco) supplemented with 10% fetal bovine serum (Hyclone-Fisher, Logan, UT) and 1% Penicillin-streptomycin (Sigma). BR04 and BR05 were culture in Dulbecco's modified eagle's medium (Cellgro, Herndon, VA), supplemented with 10% fetal bovine serum (Hyclone) and Penicillin-streptomycin (Sigma).

3D cell culture

Cell-impregnated 3D collagen matrices were prepared as described previously [1-3]. Briefly, cells suspended in a 1:1 (v/v) ratio of cell culture medium and reconstitution buffer (0.2 M 4-(2-hydroxyethyl)-1-piperazineethanesulfonic acid (HEPES) and 0.26 M $NaHCO_3$ in nanopure water) were mixed with the appropriate amounts of soluble rat tail type I collagen (BD Biosciences, San Jose, CA, USA) to achieve a final concentration of 2mg/ml. Adequate amount of 1M sodium hydroxide was added to attain a final pH of 7, after which the mixture was added to a 24-well glass bottom dish (Greiner Bio-one, NC), and immediately transferred to the incubator

maintained at physiological conditions of 37° C and 5% CO_2, to allow for collagen polymerization and cell spreading. During preparation, all ingredients were kept chilled on ice to avoid premature polymerization, with care taken to avoid the formation of bubbles during mixing. Samples were incubated overnight and were fixed and stained in preparation for image acquisition.

Immunostaining and fluorescence microscopy

Approximately 12,000 cells were plated in each well of a 24-well glass bottom plate (MatTek, MA), corresponding to approximately 20% surface coverage to ensure single cell resolution. After 16 h incubation, cells were fixed with 3.7% para-formaldehyde for 12 min at room temperature. Cells were then permeabilized with 0.1% Triton X-100 (Sigma) for 10 min; nonspecific binding was blocked with phosphate-buffered saline (PBS) supplemented with 1% albumin from bovine serum (BSA) for 40 min. Nuclear DNA was stained with Hoechst 33342 (Sigma) at 1:50 dilution, cytoplasm was stained with the non-specific dye HCS CellMask Deep red stain (Invitrogen) at 1:20000 dilution, and actin was stained with phalloidin Alexa Fluor 488 (Invitrogen) at a 1:40 dilution.

Fluorescently labeled cell samples were visualized with a Nikon digital sight DS-Qi1MC camera mounted on a Nikon TE300 epifluorescence microscope (Nikon Melville, NY), and equipped with a motorized stage and motorized excitation and emission filters (Prior Scientific, Rockland, MA) controlled by NIS-Elements (Nikon). For each sample, eighty-one (9-by-9 square grid) fields of view from a low-magnification lens (10x Plan Fluor lens; N.A. 0.3, Nikon) were used covering a contiguous area of 6.03 mm x 4.73 mm (28.5 mm^2). Three fluorescence channels for Hoechst 33342, Alexa Fluor 488 and Alexa Fluor 647 and one phase-contrast channel were recorded to obtain the necessary morphometric information about the nucleus and cellular body of every single cell within the scanning region. Segmentation of nuclear and cellular shape from images was conducted using a custom MATLAB code. Cellular and nuclear segmentation was validated using both manual tracing of cells and nuclei and using high-magnification imaging (40x Plan Fluor lens; N.A. 1.3, Nikon), as explained in the main text.

Image calibration

Unavoidable non-uniform illumination of the samples presents challenges when conducting intensity-based cell segmentation of fluorescent images. The calibration was performed using glass-bottom dish contained dyes for the different fluorescent channels. For each channel, two images were acquired: with and without illumination, which were denoted as I_F and I_B respectively.. The calibrated images, I_{CAL}, for reducing the non-uniform illumination of fluorescent images and non-uniform distribution of intensity offset was obtained from raw images, I_{RAW}, through the following equation,

$$I_{CAL}= (I_{RAW}-I_B)/(I_F-I_B) \times <I_F-I_B>$$

Calibrated images were further used for nuclei and cell segmentation and quantification (see below).

Segmentation of cellular and nuclear boundaries

To segment individual cells and nuclei, we used slightly different approaches. For nuclear segmentation, because of the relatively circular shape and relatively even intensity of the Hoechst stain, we filtered calibrated images (as described in the previous section) with a 23 x 23 pixel normalized Gaussian filter (similar scale as the size of nuclei) and a same size of averaging filter same size to obtain Gaussian intensity, I_G, and average intensity, I_M,. Subtracting I_M from I_G gives I_N, the nuclear intensity values without regional background. Empirical testing showed that a threshold setting of 10 was optimal.

Images of cells were first processed with a 3x3 averaging smoothing filter to reduce noises. Proper estimation of background intensity level is critical to threshold cell boundaries accurately. Most fields of view in an image are cell-free; therefore, large portions of pixels reflect background intensity. The background intensity was characterized by the mode value and standard deviation of these pixels. Here, we adopted an iterative process for robust background intensity distribution. Briefly, we give an intensity threshold value, I_{TH}, to find a subset of pixels, I_S, with intensity value that is less than this intensity threshold. The intensity threshold value for the next iteration is then updated using the following equation,

$$I_{TH} = I_{BG} + 3.5 \times I_{RBG},$$

where I_{BG} is the most frequent intensity among I_S and I_{RBG} is the standard deviation of I_S. The first threshold value was set using the maximum intensity of image. Three to five iterations

generally resulted in stable values of I_{BG} and I_{RBG}, which represent the average background intensity value and associated noise in background intensity magnitude, respectively. Next, we used I_{BG} and I_{RBG} to select the signal region of fluorescently labeled cells. We defined the threshold factor, th_{cell} to select all the pixels in the image with an intensity value larger than a threshold intensity($=I_{BG} + th_{cell} \times I_{RBG}$). The value for th_{cell} was optimized by empirical observation, which was usually between 2 and 5.

Implementing the above approach, we determined cell boundaries using phalloidin-stained F-actin images. F-actin usually gives a stronger signal at the cell boundary than at the cell center, differentiating the boundary from the cytoplasm with less bias than a more homogenous dye (such as HCS cell mask). In contrast, HCS cell mask intensities concentrated around the nucleus - the thicker region of the cell - and decayed towards the edge of the cell; because of the low NA objective, the edge intensity values was blurred, making edge detection very sensitive to bias and sample-to-sample variations.

However, this method only worked for isolated cells. When cells were very close to one another or in direct contact, edge intensity values given by phalloidin staining were all above background and did not allow for direct segmentation. In these cases, we could use HCS cell mask to perform watershed segmentation and identify edges between contacted cells. Then collected set of nuclei and cell objects were used to calculate their associated morphological descriptors, as described in the next section. Overall, the more than 95% of cells are segmented accurately based on manual inspection. The shape factor (*SF*) of a shape is defined as $4\pi A/P^2$, where A and P is the area and perimeter of a shape.

Decomposition of 2-dimensional shape and identification of shape mode

Alignment of cell shapes and nucleus shapes was implemented using Procrustes analysis [4, 5]. In brief, after the boundary coordinates of each segmented cellular or nuclear shape in an image were obtained, they were resampled to 50 positions that divided the boundaries evenly. The boundary coordinates were then subtracted by their mean value so that the center of the object was located at the coordinate (0, 0). The scale of a shape (R) was calculated using the normalized coordinates, $Z = (x_i, y_i)$, through the following equation,

$$R = \sqrt{\sum_{i=1}^{50}(x_i^2 + y_i^2)/50}.$$

To unify the scale, the boundaries coordinates of a shape was further normalized by dividing it by the scale (R). To eliminate the variation of shape due to rotational or mirror effects, we first aligned the major axis of a shape with the horizontal axis by applying a rotational matrix (V_M) to the boundaries coordinates. The rotational matrix was obtained from the singular vector decomposition of Z, where $Z = U_M S_M V_M^T$. The average shape Z_R was obtained by averaging the normalized boundaries coordinates of all cells from different cell lines. For each cell shape, a rotational matrix Q that minimized the distance between Z_R and Z, i.e.,

$$E = (QZ - Z_R)(QZ - Z_R)^T,$$

was obtained from the singular value decomposition of $Z_R^T Z = U_R S_R V_R$ and Q is the matrix product VU^T. Due to the fact that cellular and nuclear shapes are enclosed objects, each of the 50 coordinates were used as a starting point in either counterclockwise or clockwise directions and the corresponding linear sets were examined to identify the sequence that yielded minimization of E for each shape. Coordinates from this aligned shape were used as descriptors for the shape. Principal component analysis (PCA) was then applied on these descriptors for all cell samples to obtain eigenshape vectors. The principal components from the eigenshape vectors that spanned 95% of total variance were used as simplification set of descriptors for cellular or nuclear shapes. K-means clustering analysis was then implemented to identify the shape subtypes, i.e. shape modes, based on these principal components descriptors. The number of shape modes was identified based on separation index [6] and Xie and Beni index [7].

Determination of the phases of the cell cycle for each single cell

DNA content of each nucleus was estimated by integrating nuclear intensity of Hoechst 33342 labeled DNA [8]. Histograms of DNA content for each cell lines revealed the distribution of the cell cycle phases. To determine the percentage of cells in each of the cell-cycle phase, i.e. G1 phase, S phase and G2/M phase, we fit the DNA content distribution using the Dean Jett polynomial model [9]. Therefore, at a given value of DNA content from a cell we estimated its phase at the cell cycle.

Determination of the cell clustering or singlet status

For each scanned cell sample, the locations of nuclei on the motorized stages (x_s, y_s) were estimated from the stage location of the image field, (x_{IS}, y_{IS}), and cell locations in the image field, (x_I, y_I), through the following equations;

$$(x_s, y_s) = (x_{IS}, y_{IS}) + px \times (x_I, y_I)$$

, where *px* is the pixel size of image. Hence, the distance of a cell to the nearest neighbor cell was measured. Average radius of pancreatic epithelium cells is approximately 25 µm and hence if cell has no direct physical contacts with other cells, i.e. the singlet cells, the estimated least distance to the nearest cells singlet cells condition are twice the cell size (=50 µm). The singlet cell condition was estimated based on the cell with the nearest distance to next cells more than 50 µm. The clustering cells are the cells do not fulfill this criterion.

Sub-sample cross-validation for prediction accuracy

In addition to the blind tests described in the main text, to measure predictive accuracy, we used a repeated random sub-sampling cross-validation strategy based on increasing number of cells. We first randomly selected 9 cell lines among 11 PT or metastasis cell lines and build a classifier using generalized linear model to predict the metastasized status of cells lines using single morphology feature, *X*. We used Binomial distribution with Logit link function to establish generalized linear model model, i.e.

$$Y=\exp(X\beta)/(1+\exp(X\beta)).$$

Where Y is predicted probability of being metastasized cell lines and β is fitted model parameters. We then applied this classifier to the other 2 cell lines which are represented by the randomly selected cell sample with specified sample size. The process was then repeated 200 times to estimate overall sensitivity, specificity, and accuracy. The overall accuracy of cross-validation results is measured by (TP+TN)/(TP+FP+FN+TN), where TP is true positive counts, TN is true negative counts, FP is false positive counts and FN is false negative counts. Sensitivity and specificity is estimated TP/(TP+FN) and TN/(TN+FP) respectively.

Analysis of cell morphology heterogeneity

The Shannon's entropy, S, of nucleus and cell shape mode (SM) distribution is calculated using the following equation,

$$S = \sum_i P(SM_k) \cdot logP(SM_k)$$

, where $P(SM_k)$ is the fraction of cells exhibit k-th shape mode, SM_k. Variance of morphology parameters including nucleus size and cell size, were calculated by $\sigma_M^2 = <(M - <M>)^2>$, where M is the morphology parameters and <> is expected value of given variables. Variance of cell shapes and nucleus shape were calculated by $\Sigma\ \sigma_{csk}^2$ and $\Sigma\ \sigma_{nsk}^2$ where csi or nsi denoted the projected scores at i-th eigenshape factors. To be noted, only the eigenshape vectors that spanned 95% of total variance were used.

Supplementary figure captions

Supplementary figure 1. **Automated cell segmentation: comparison with manual segmentation**. **A**. Cellular and nuclear segmentation of four representative cells using the automated software approach used in this work (purple) and manual tracing (blue). **B**. Segmentation of the same 100 randomly selected cells using both methods. Results show that boundaries of cells obtained from the automated segmentation process correspond well with manually traced boundaries of cell and nucleus. **C**. The same cells were imaged using a high N.A (1.45), high magnification (60X) objectives and using a low N.A (0.3), low magnification (10X) objective. Automated segmentation of the same cells and nuclei was performed using both objectives. Cell and nuclear traces from 100 randomly selected cells are shown. The well corresponding cell and nucleus traces from both setups suggest the low N.A and low magnification objective yield the sufficient segmentation resolutions.

Supplementary figure 2. **Evaluation of optimum nuclear and cellular shape subtypes (cluster number) using the separation index (S) and the Xie and Beni's index (XB)**. The optimum number of clusters should minimize the value of the index. Here, nuclear shapes and cellular shapes are parameterized by their value at a set of previous identified eigenshape vectors. K-means clustering analysis was applied to nucleus shape samples and cell shapes sample using different cluster number and S index and XB index were calculated for each condition. **A-B**. Plots show the S index (**A**) and XB index (**B**) as function of cluster numbers (shape subtypes)

from nucleus shapes. **C-D**. Plots show the S index (**C**) and XB index (**D**) as function of cluster numbers (shape subtypes) using cells shapes.

Supplementary figure 3. Robustness of the VAMPIRE analysis. To ensure the repeatability and reliability of the identification of shape modes using this quantitative assay, different preparation of the PAC004 cell samples were imaged and their nucleus shapes were collected. The eigenshape vectors for nucleus which were identified previously from all pancreatic cancer cell lines were applied to decompose a nucleus shape k in to a vectors P_k in which the vector components represent the projection value at various eigenshape vectors. Individual shape modes as previously identified can also be represented by the same sets of vector (P_S). The shape mode which has minimum paired distance to the P_k were the designated shape mode for the nucleus shape k. Thus, for each obtained nucleus shapes a nucleus shapes mode were assigned and nuclear shape mode distribution of different experiments was obtained. **A**. The very consistent nuclear shape mode distribution was observed in all experiment replicates cell samples and is users independent. **B**. Nuclear shape mode distributions from samples being imaged at different days after fixation and fluorescent labeling show a conserved trend.

Supplementary figure 4. Hierarchy clustergram of nucleus shape mode composition for PDAC cells. A clustergram shows the probability of cells in each nucleus shape modes ($P(NS_k)$) for different PDAC cell lines. Color coding (red to green) corresponds to low and high occurrence. Hierarchy clustering results show that several PDAC cells display similar nucleus shape mode distributions, PAC05, PAC06, PAC07; PAC08, PAC09, PAC10; and PAC04, PAC03 respectively. PAC05, PAC06 and PAC07 have similar compositions of nucleus shape modes.

Supplementary figure 5. Morphological properties of PDAC cells. A-E. The scatter plot shows the distribution of nuclear shape entropy (A), cell shape mode entropy (B), paired nucleus-cell shape mode entropy (C), CV of nucleus size (D), CV of cell size (E) for LM samples and PT samples. **F-G**. Coefficient of variation of the shape factors for LM and PT samples are plotted. **H-I**. Average nucleus size (H) and cell size (I) is also insignificant different

between LM samples and PT samples. The reported *P*-values were calculated based on two sample *t*-test.

Supplementary figure 6. Number of gene alterations in pancreatic cancer. A dot plot shows the number of gene alterations for each PT and LM cell lines. No significant difference ($P > 0.4$) was found between PT and LM cell lines using two sample *t*-test.

Supplementary figure 7. **Heterogeneity breast cancer cell morphology. A**. A heatmap shows the degree of morphological heterogeneity of breast cancer cell lines, including 4 lines derived from primary tumors at disease stage IIB (PT) and 6 lines derived from metastatic site (M) at disease stage IV (see detailed information of cell lines in **Supplementary Table 3**). **B**. A 3-D scatter plot shows CV of nuclear size, CV of cell size and entropy of cell shape of PT (circle) and M (square) of breast cancer cell lines. The cell lines with ER/PR/HER2 negative (TN) are highlighted (+). **C and D**. Plots show The CV of nuclear size (C) and shape entropy (D) for breast cancer cell lines derived from metastatic lesion (M) and primary tumors (P). Nuclear size and nuclear shape entropy is positively correlated with decrease of tumor stage (i.e. metastatic or primary tumor), and Pearson correlation coefficients (ρ) of 0.33 and 0.20. No significant difference was found ($P>0.05$). **E and F**. The CV of cellular size (E) and shape entropy (F) for breast cancer cell lines derived from metastatic lesion (M) and primary tumors (PT) were shown. The Pearson correlation coefficient (ρ) for cellular size and shape entropy with tumor stages is 0.42 and 0.42. No significant difference was found ($P>0.05$). The reported *P*-values were calculated based on two sample *t*-test.

Supplementary figure 8. **A predictive model for metastatic pancreatic cancer cells. A**. CV cellular size distribution of singlet cells was used as predictor of metastatic characteristic for a cell line. Accuracy, specificity and sensitivity as a function of sample size are shown in the plot. Overall accuracy reaches 95% when using ~250 singlet cells. **B and C**. The same procedure was applied using the CV of nuclear size distribution and the entropy of nuclear shape as predictors. Overall accuracy reaches 95% with a sample size of ~350 cells.

Supplementary figure 9. **Hierarchy of cell morphological heterogeneity. A**. Bar graphs show the average variances of nucleus size and cell size among different pancreatic cancer cells at different underlying cellular conditions after random permutation of cells in each experiment. Variation in nucleus size and cell size is independent of cellular condition after random permutation. **B**. Bar graphs show the average variances of nucleus shape and cell shape among different pancreatic cancer cells at different underlying cellular conditions. The shape variances are measured by total variance among projection scores in eigenshape vectors and normalized by the variance among all populations. Great decrease in variations in nucleus shapes was found for clonal cells at G0/G1 phase. **C**. Variance of averaged nucleus shape and averaged cell shape among different progenies are used to measure heritable variation for both LM (PAC01~PAC04) and PT (PAC06~ PAC09). LM display in average lower heritable variation in both nucleus shape and cell shape ($P > 0.05$). The reported *P*-values were calculated using two sample *t*-test.

Supplementary tables

Supplementary Table 1. List of the patient-derived pancreatic cancer cells line used in this study.

Supplementary Table 2. List of the breast cancer cells line used in this study.

Supplementary Table 3. Correlation coefficients of heterogeneity parameters between PT cells and LM cells in different conditions. NC=nuclear-cell; N=nuclear; C=cell; pop=population; sc=scale; cv=coefficient of variation.

References

1. Wu, P.H., Giri, A., Sun, S.X. & Wirtz, D. Three-dimensional cell migration does not follow a random walk. *P Natl Acad Sci USA* **111**, 3949-3954 (2014).
2. Fraley, S.I. et al. A distinctive role for focal adhesion proteins in three-dimensional cell motility. *Nat Cell Biol* **12**, 598-604 (2010).
3. Giri, A. et al. The Arp2/3 complex mediates multigeneration dendritic protrusions for efficient 3-dimensional cancer cell migration. *Faseb J* **27**, 4089-4099 (2013).
4. Bookstein, F.L. Landmark methods for forms without landmarks: Localizing group differences in outline shape. *Proceedings of the Ieee Workshop on Mathematical Methods in Biomedical Image Analysis*, 279-289 (1996).
5. Dryden, I.L.M., I.L.D Statistical Shape Analysis. (John Wiley, 1998).
6. Bensaid, A.M. et al. Validity-guided (re)clustering with applications to image segmentation. *Ieee T Fuzzy Syst* **4**, 112-123 (1996).
7. Xie, X.L.L. & Beni, G. A Validity Measure for Fuzzy Clustering. *Ieee T Pattern Anal* **13**, 841-847 (1991).
8. Wu, P.H., Hung, S.H., Ren, T.N., Shih, I.M. & Tseng, Y. Cell cycle-dependent alteration in NAC1 nuclear body dynamics and morphology. *Phys Biol* **8** (2011).
9. Dean, P.N. & Jett, J.H. Mathematical-Analysis of DNA Distributions Derived from Flow Microfluorometry. *J Cell Biol* **60**, 523-527 (1974).

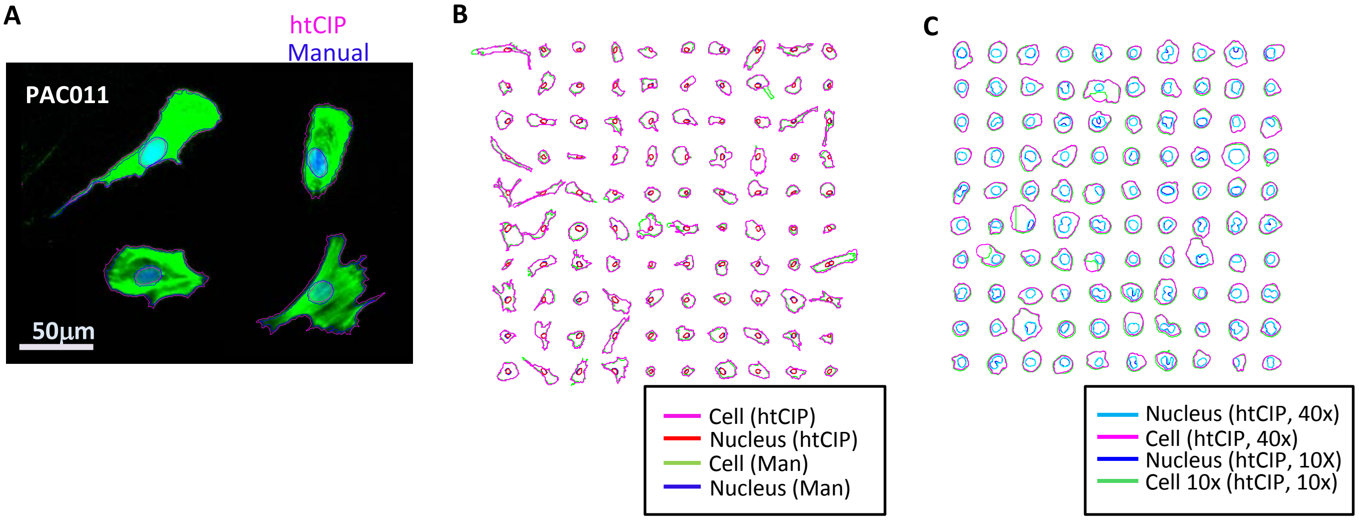

Supplementary Figure 1. Wu et al.

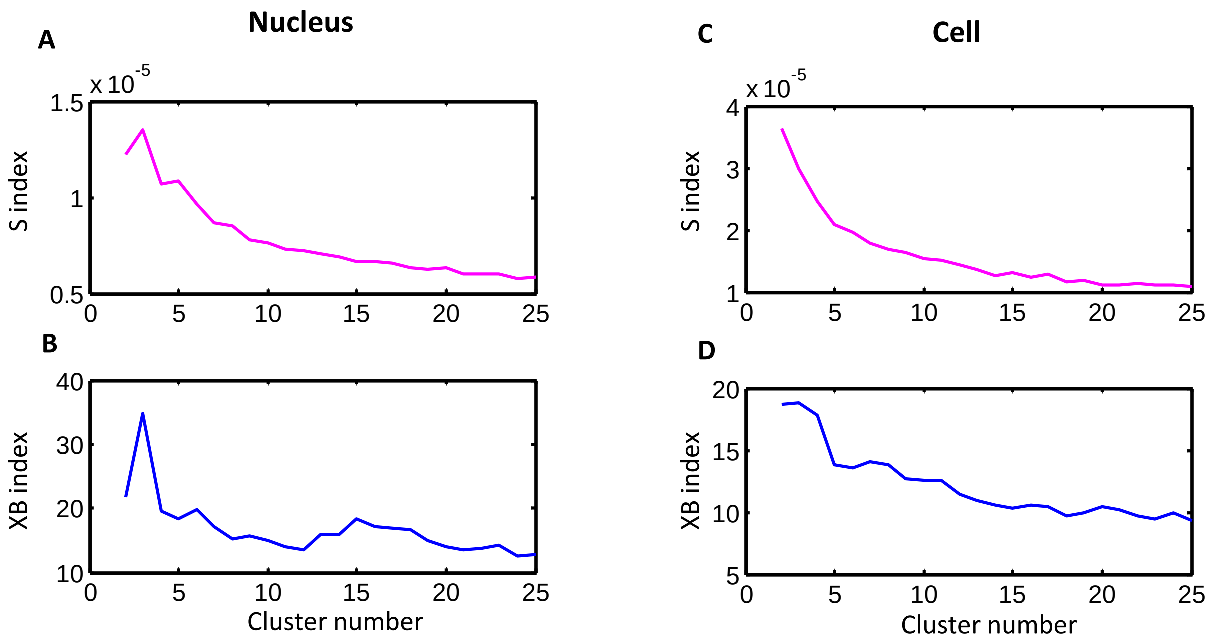

Supplementary Figure 2. Wu et al.

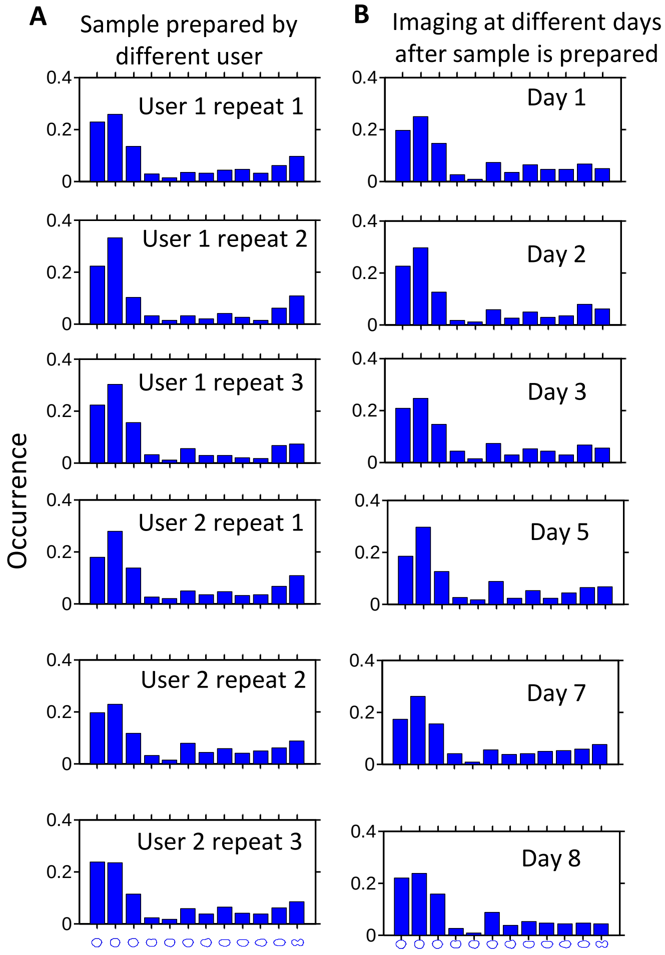

Supplementary Figure 3. Wu et al.

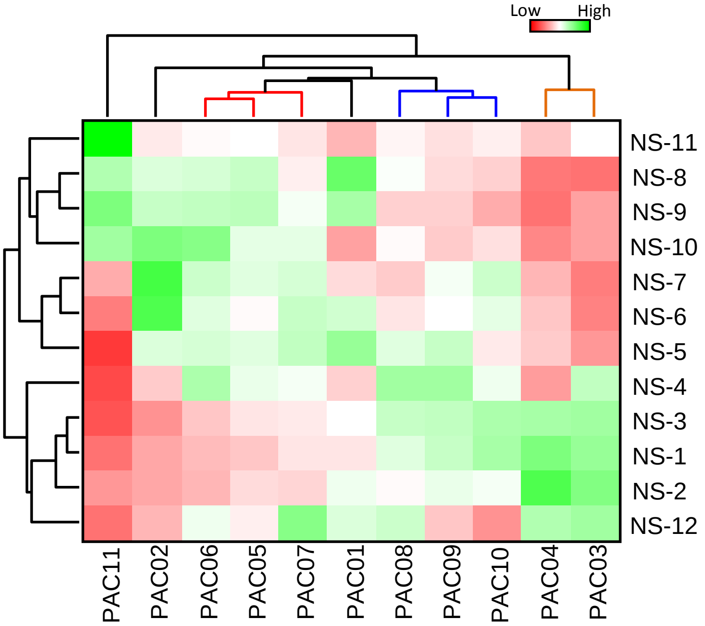

Supplementary Figure 4. Wu et al.

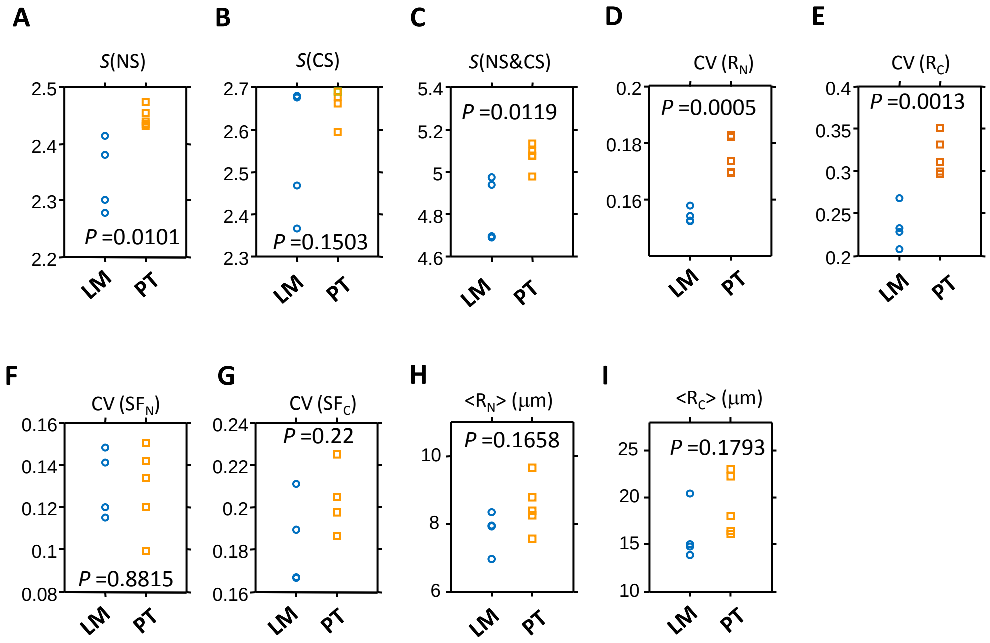

Supplementary Figure 5. Wu et al.

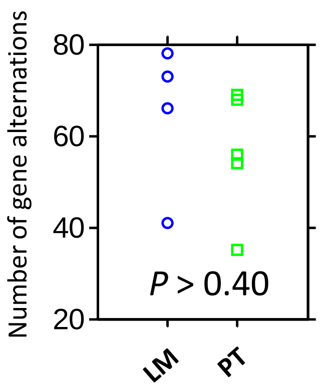

Supplementary Figure 6. Wu et al.

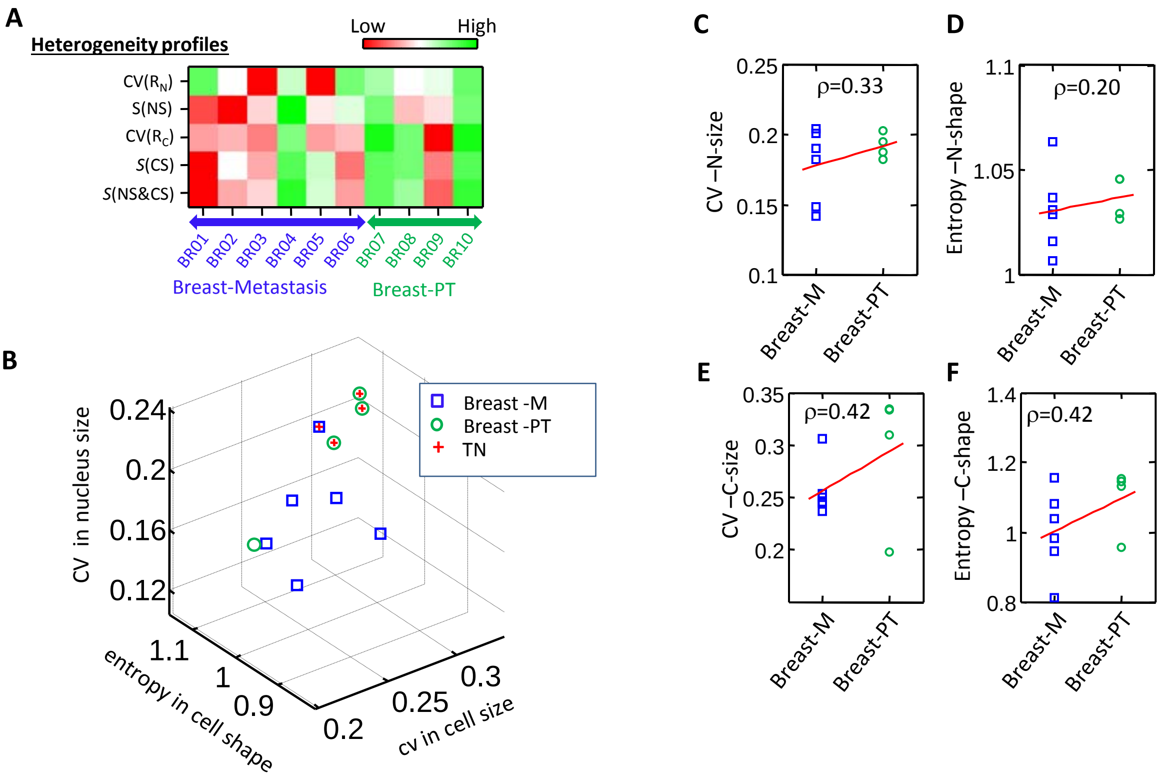

Supplementary Figure 7. Wu et al.

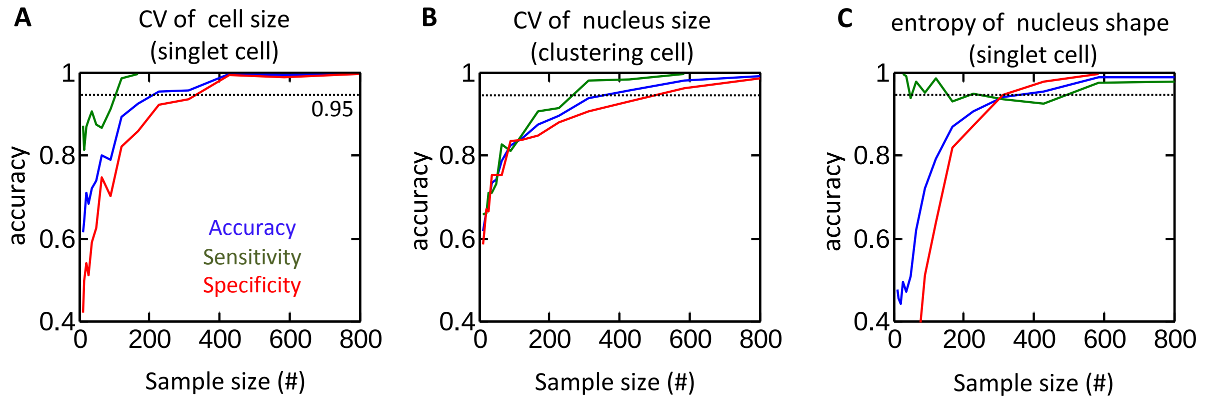

Supplementary Figure 8. Wu et al.

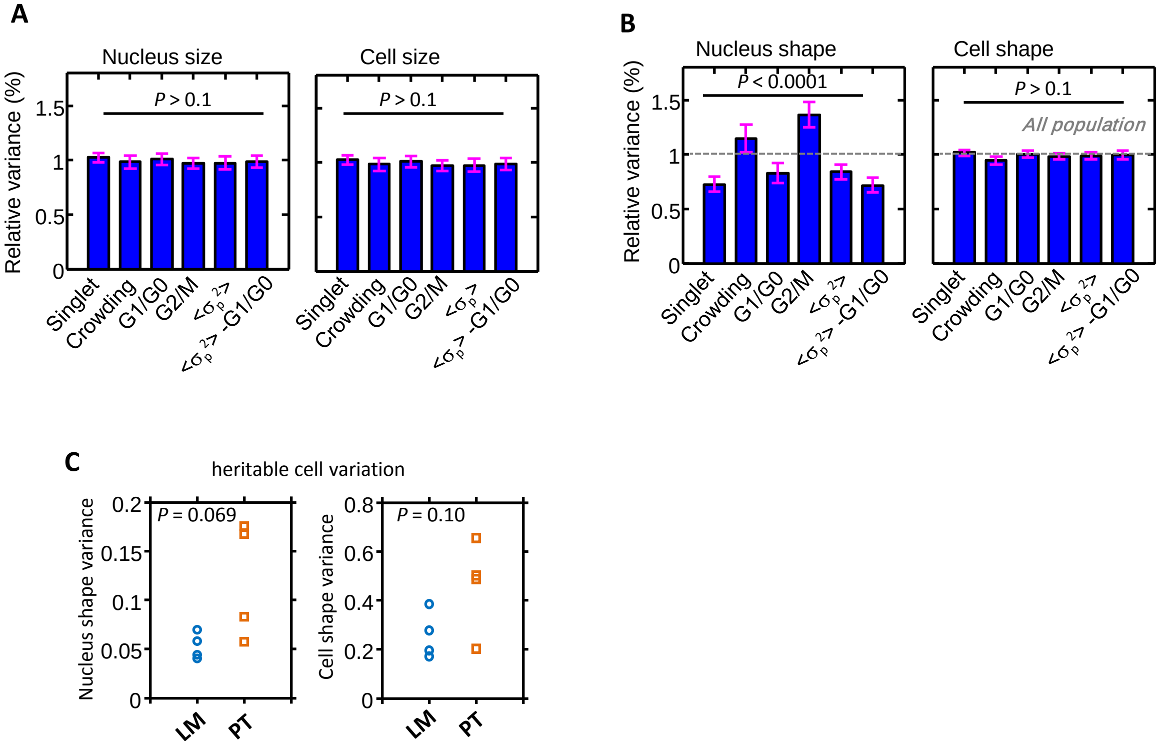

Supplementary Figure 9. Wu et al.

Sample	Official name	Other name	Age	Sex	Disease type	Tissue derivation	Stage
PAC01	A2.1	Pa01C	62	M	Ductal adenocarcinoma	Liver metastasis	IV
PAC02	A38.5	Pa08C	51	M	Ductal adenocarcinoma	Liver metastasis	IV
PAC03	A6L	Pa02C	57	M	Ductal adenocarcinoma	Liver metastasis	IV
PAC04	A10.7	Pa03C	60	M	Ductal adenocarcinoma	Liver metastasis	IV
PAC05	Panc215	Pa09C	60	F	Ductal adenocarcinoma	Primary pancreatic tumor	IIB
PAC06	Panc10.05	Pa16C	81	M	Ductal adenocarcinoma	Primary pancreatic tumor	IIB
PAC07	Panc198	Pa20C	69	M	Ductal adenocarcinoma	Primary pancreatic tumor	IIB
PAC08	Panc2.5	Pa21C	54	F	Ductal adenocarcinoma	Primary pancreatic tumor	IIB
PAC09	Panc5.04	Pa18C	77	F	Ductal adenocarcinoma	Primary pancreatic tumor	IIB
PAC10	HPDE	HPDE-6/E6E7	63	F	Normal ductal epithelium	Normal Pancreas	N/A
PAC11	HPNE		52	M	Normal epithelium	Normal Pancreas	N/A
PAC20	JD13D	Pa04c	59	M	Ductal adenocarcinoma	Lung metastasis	IV
PAC21	3.014	Pa28c	65	M	Ductal adenocarcinoma	Primary pancreatic tumor	IIB

Supplementary Table 1. Wu et al.

Sample	Official name	Other name	Age	Sex	ER	PR	Her2	Disease type	Tissue derivation	Stage
BR01	ZR-75-1	ATCC CRL-1500	63	F	+	-	-	Ductal carcinoma	ascites fluid metastasis	IV
BR02	T47D	ATCC CRL-2865	54	F	+	+	-	Ductal carcinoma	Pleural effusion metastasis	IV
BR03	Cama1	ATCC HTB-21	51	F	+	-	-	Adenocarcinoma	Pleural effusion metastasis	IV
BR04	MDA231	ATCC HTB-26	51	F	-	-	-	Adenocarcinoma	Pleural effusion metastasis	IV
BR05	HCC1428	ATCC CRL-2327	49	F	+	+	-	Adenocarcinoma	Pleural effusion metastasis	IV
BR06	HCC1569	ATCC CRL-2330	70	F	-	-	+	Metaplastic carcinoma	Mammary gland	IV
BR07	HCC38	ATCC CRL-2314	50	F	-	-	-	Ductal carcinoma	Mammary gland; breast/duct	IIB
BR08	HCC1806	ATCC CRL-2335	60	F	-	-	-	Acantholytic squamous cell carcinoma	Mammary gland	IIB
BR09	HCC1500	ATCC CRL-2329	32	F	+	+	-	Ductal carcinoma	Mammary gland; breast/duct	IIB
BR10	HCC1937	ATCC CRL-2336	23	F	-	-	-	Ductal carcinoma	Mammary gland; breast/duct	IIB

Supplementary Table 2. Wu et al.

Heterogeneity parameters	overall	cell-cell contact		cell cycle		
		clustering	singlet	G0/G1	S	G2/M
CV of nucleus size	*0.91 [0.64 0.98]*	*0.93 [0.69 0.99]*	*0.79 [0.26 0.95]*	*0.56 [-0.17 0.89]*	*0.80 [0.28 0.96]*	*0.61 [-0.09 0.90]*
Nucleus shape entropy	*0.79 [0.28 0.96]*	*0.84 [0.40 0.97]*	*0.83 [0.37 0.96]*	*0.68 [0.03 0.93]*	*0.88 [0.51 0.97]*	*0.44 [-0.32 0.85]*
CV of cell size	*0.89 [0.55 0.98]*	*0.81 [0.31 0.96]*	*0.90 [0.59 0.98]*	*0.83 [0.37 0.96]*	*0.84 [0.39 0.96]*	*0.76 [0.20 0.95]*
Cell shape entropy	*0.52 [-0.22 0.88]*	*0.55 [-0.18 0.89]*	*0.59 [-0.11 0.90]*	*0.51 [-0.24 0.88]*	*0.59 [-0.12 0.90]*	*0.52 [-0.22 0.88]*
NC pop entropy	*0.79 [0.26 0.95]*	*0.89 [0.56 0.98]*	*0.66 [0.00 0.92]*	*0.75 [0.16 0.94]*	*0.80 [0.28 0.96]*	*0.75 [0.16 0.94]*

Note: [] represents lower and upper bounds for a 95% of confidence interval for each coefficient

Supplementary Table 3. Wu et al.
